# P-1517. Adult Respiratory Syncytial Virus Vaccination Receipt among Adults Testing Negative for RSV – VISION Network, October 1, 2024—March 31, 2025

**DOI:** 10.1093/ofid/ofaf695.1701

**Published:** 2026-01-11

**Authors:** Morgan Najdowski, Josephine Mak, Cassandra A Hathaway, Patrick K Mitchell, Angela Cheung, Gabriela Vazquez-Benitez, Kristin K Dascomb, Stephanie Irving, Nicola P Klein, Shaun J Grannis, Toan Ong, Adriana V Resendez, Sarah W Ball, Jingran Cao, Charlene E McEvoy, Tamara Sheffield, Daniel Bride, Julie Arndorfer, Joshua Van Otterloo, Allison L Naleway, Ousseny Zerbo, John R Hansen, Lawrence Block, Karen B Jacobson, Brian E Dixon, Colin Rogerson, Thomas Duszynski, William F Fadel, Michelle Barron, David Mayer, Catia Chavez, Karthik Natarajan, Amber Kautz, Allison Avrich Ciesla, Amadea Britton, Ndey Bassin Jobe, Jennifer DeCuir, Ryan E Wiegand, Amanda B Payne, Ruth Link-Gelles

**Affiliations:** CDC, Atlanta, Georgia; Division of Healthcare Quality Promotion, Centers for Disease Control and Prevention, Atlanta, Georgia; Westat, Rockville, Maryland; Westat, Rockville, Maryland; Westat, Rockville, Maryland; HealthPartners Institute, bloomington, Minnesota; Intermountain Healthcare, Murray, Utah; Kaiser Permanente Center for Health Research, Portland, Oregon; Division of Research Kaiser Permanente Vaccine Study Center, Oakland, California; Indiana University, Indianapolis, Indiana; University of Colorado Anschutz Medical Campus, Centennial, Colorado; Westat, Rockville, Maryland; Westat, Rockville, Maryland; HealthPartners Institute, bloomington, Minnesota; HealthPartners Institute, bloomington, Minnesota; IntermountainHealth, Salt Lake City, Utah; Intermountain Healthcare, Murray, Utah; Intermountain Healthcare, Murray, Utah; IntermountainHealth, Salt Lake City, Utah; Kaiser Permanente Center for Health Research, Portland, Oregon; Division of Research Kaiser Permanente Vaccine Study Center, Oakland, California; Division of Research Kaiser Permanente Vaccine Study Center, Oakland, California; Kaiser Permanente Northern California, Oakland, California; Kaiser Permanente Vaccine Study Center, Oakland, California; Regenstrief Institute, Indianapolis, Indiana; Regenstrief Institute, Indianapolis, Indiana; Regenstrief Institute, Indianapolis, Indiana; Regenstrief Institute, Indianapolis, Indiana; University of Colorado, Aurora, Colorado; University of Colorado Anschutz Medical Campus, Centennial, Colorado; University of Colorado, Aurora, Colorado; Columbia University, New York, New York; Centers for Disease Control and Prevention, Atlanta, Georgia; Centers for Disease Control and Prevention, Atlanta, Georgia; Centers for Disease Control and Prevention, Atlanta, GA; Centers for Disease Control and Prevention, Atlanta, Georgia; Centers for Disease Control and Prevention, Atlanta, Georgia; Centers for Disease Control and Prevention, Atlanta, Georgia; CDC, Atlanta, Georgia; Centers for Disease Control and Prevention, Atlanta, Georgia

## Abstract

**Background:**

On June 26, 2024, the CDC updated respiratory syncytial virus (RSV) vaccine recommendations to a single dose of RSV vaccine for all adults aged ≥ 75 years and adults aged 60-74 years with increased risk of severe RSV disease. Using electronic health record (EHR) data from the VISION platform, we described characteristics of patients testing negative for RSV who did and did not receive an RSV vaccine and assessed factors associated with RSV vaccine receipt.

**Figure 1:**
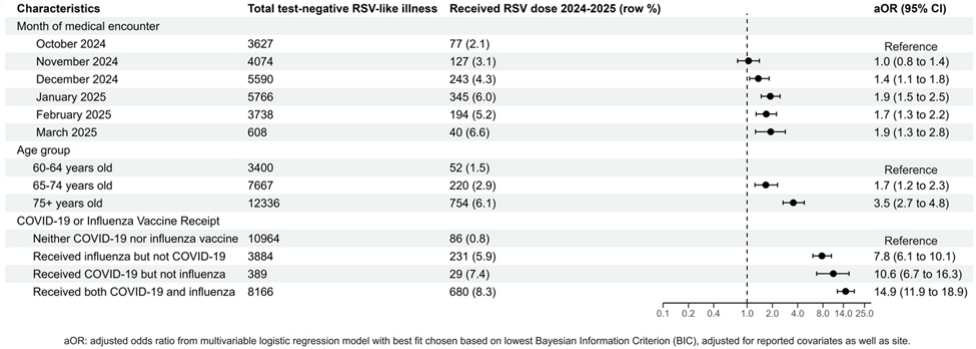
Characteristics associated with receipt of respiratory syncytial virus (RSV) vaccination among test-negative patients with an emergency department (ED) encounter for RSV-like illness (RLI) during the 2024-2025 season in the VISION network, N=23,403 patients

**Figure 2:**
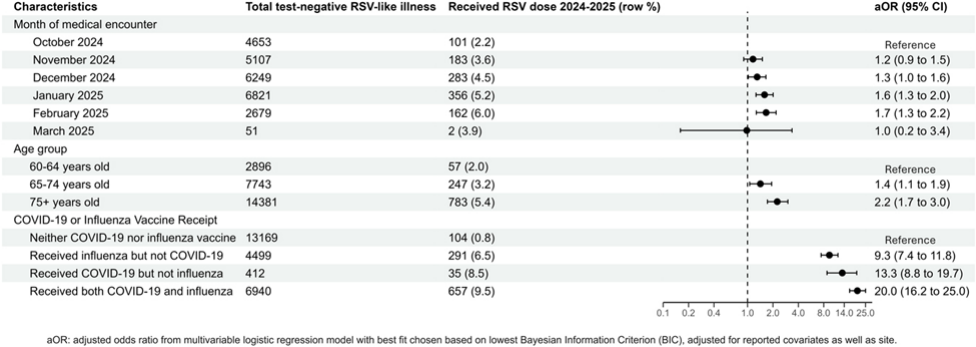
Characteristics associated with receipt of respiratory syncytial virus (RSV) vaccine among test-negative patients with an inpatient encounter for RSV-like illness (RLI) during the 2024-2025 season in the VISION network, N=25,020 patients

**Methods:**

Patients with ≥ 1 emergency department (ED) or inpatient encounter at any of 6 participating health systems in 8 states with RSV-like illness (RLI) during October 1, 2024-March 31, 2025 were included. Vaccination status was ascertained from EHR, state and city immunization information systems, and medical claims. Patients who tested positive for SARS-CoV-2 or influenza viruses at the same RLI encounter were excluded. Patient age, sex, race and ethnicity, Medicaid status, number of underlying medical conditions, month of medical encounter, and documented receipt of COVID-19 or influenza vaccines were evaluated as covariates when assessing the odds of vaccination. The best fitting multivariable logistic regression models using Bayesian Information Criterion were chosen.

**Results:**

Among 48423 included patients, 2113 (4.4%) had documented RSV vaccine receipt. The odds of RSV vaccination differed by site and increased with calendar time and age. Compared to patients aged 60-64 years, those aged ≥ 75 years were more likely to have received an RSV vaccine (ED: aOR: 3.6, 95%CI: 2.7-4.8, Figure 1; inpatient: aOR: 2.3, 95%CI: 1.7-3.0, Figure 2). Receipt of both influenza and COVID-19 vaccine within the same season had the strongest association with RSV vaccination in both the ED (aOR: 14.88, 95%CI: 11.87-18.89, Figure 1) and hospital setting (aOR: 20.04, 95%CI: 16.20-25.03, Figure 2).

**Conclusion:**

Receipt of other respiratory viral vaccines was the strongest indicator of RSV vaccination in the 2024-2025 RSV season in patients testing negative for RSV among all demographic and clinical characteristics considered. RSV vaccination was lower among those aged 60-64 years than older patients. These findings inform future methods to estimate vaccine effectiveness and inform policy implementation.

**Disclosures:**

Gabriela Vazquez-Benitez, PhD, MSc, AbbVie: research funding not related to this study|Sanofi: Grant funding for other research not related to this study Stephanie Irving, MHS, Westat: Grant/Research Support Nicola P. Klein, MD, PhD, AstraZeneca: Grant/Research Support|Centers for Disease Control and Prevention: Grant/Research Support|GlaxoSmithKline: Grant/Research Support|Janssen: Grant/Research Support|Merck: Grant/Research Support|Moderna: Grant/Research Support|Pfizer: Grant/Research Support|Sanofi Pasteur: Grant/Research Support|Seqirus: Grant/Research Support Shaun J. Grannis, MD, MS, Centers for Disease Control and Prevention: Grant/Research Support|National Institutes of Health NCATS: Grant/Research Support|National Institutes of Health NIMH: Grant/Research Support Toan Ong, PhD, Centers for Disease Control and Prevention via Westat: Grant/Research Support|Patent Title: Systems and Methods For Record Linkage: Patent Number: PCT/US2018/047961|PCORI: Travel Support|Regenstrief Institute: Advisor/Consultant|Regenstrief Institute: Travel Support Sarah W. Ball, MPH, ScD, Centers for Disease Control and Prevention, Contract #200-2019-F-06819: Grant/Research Support|Centers for Disease Control and Prevention, Contract #75D30121D12779: Grant/Research Support|Novavax: Grant/Research Support Jingran Cao, MS, Sanofi Pasteur: Grant/Research Support Charlene E. McEvoy, MD, MPH, Astra Zeneca: Grant/Research Support|Centers for Disease Control and Prevention: Grant/Research Support|Department of Defense: Grant/Research Support|GlaxoSmithKline: Grant/Research Support|National Institutes of Health: Grant/Research Support|PCORI: Grant/Research Support Ousseny Zerbo, PhD, Centers for Disease Control and Prevention: Grant/Research Support|Moderna: Grant/Research Support|National Institutes of Health: Grant/Research Support|Pfizer: Grant/Research Support John R. Hansen, MPH, Centers for Disease Control and Prevention: Grant/Research Support Lawrence Block, MPH, MPA, Centers for Disease Control and Prevention: Grant/Research Support Karen B. Jacobson, MD, MPH, Centers for Disease Control and Prevention: Grant/Research Support|National Institutes of Health: Grant/Research Support|Pfizer: Grant/Research Support William F. Fadel, PhD, Centers for Disease Control and Prevention: Grant/Research Support Catia Chavez, MPH, Westat: Grant/Research Support Karthik Natarajan, PhD, Centers for Disease Control and Prevention: Grant/Research Support Ryan E. Wiegand, PhD, Merck & Co., Inc.: Stocks/Bonds (Public Company)|Sanofi S.A.: Stocks/Bonds (Public Company)

